# Insomnia in the elderly: reported reasons and their associations with medication in general practice in Denmark

**DOI:** 10.1080/02813432.2020.1753382

**Published:** 2020-05-02

**Authors:** Ida Hollsten, Billa Mouritsardóttir Foldbo, Merethe Kristine Kousgaard Andersen, Jørgen Nexøe

**Affiliations:** Research Unit of General Practice, University of Southern Denmark, Odense, Denmark

**Keywords:** Insomnia, elderly, older adults, drug prescription, hypnotics, benzodiazepines, general practice

## Abstract

***Objective:*** The aim of this study was to investigate reasons for insomnia symptoms and their associations with sleep medication prescription in elderly patients in general practice.

***Design:*** Over a period of 20 weekdays, general practitioners (GPs) recorded reasons and treatment for insomnia symptoms. Patient characteristics and outcomes were analysed using descriptive statistics. Logistic regression was used to analyse the associations between reasons for insomnia symptoms and prescription.

***Setting:*** General practices in the Region of Southern Denmark.

***Subjects:*** Consultations (*n* = 405) with patients older than 65 years presenting with insomnia symptoms.

***Main outcome measures:*** Reasons for insomnia symptoms and sleep medication prescription.

***Results:*** The most commonly reported reasons for insomnia symptoms were somatic illness (34%) and psychiatric diagnosis (29%). Having a psychiatric diagnosis or multiple reported reasons for insomnia increased the odds for prescription (odds ratio (OR) 4.60, 95% confidence interval (CI) 2.41-9.90 and OR 2.10, CI 1.03-4.28), whereas being first consultation regarding insomnia symptoms decreased the odds (OR 0.17, CI 0.10-0.30). A total of 80% received a prescription, most frequently of Z-hypnotics (49%). About half (52%) of the patients consulting their GP for the first time with insomnia symptoms received a prescription.

***Conclusion:*** Somatic and psychiatric diseases were the most commonly reported reasons for insomnia symptoms in the elderly, suggesting a high prevalence of comorbid insomnia. Regardless of reason, a majority of the consultations resulted in prescription of sleep medication with potential serious adverse effects. This indicates that there is still room for improving the management of insomnia among older adults.

Key Points

Although insomnia is common in the elderly, little is known about its reasons and their associations with prescription patterns.

-   The most commonly reported reasons for insomnia symptoms in the elderly are psychiatric diagnosis and somatic illness.
-   According to guidelines, sleep medication with potential serious adverse effects is prescribed too frequently to elderly patients.
-   An effort should be made to identify and optimally treat comorbid insomnia, which appears to be prevalent in older adults.

## Introduction

Difficulties falling asleep, interrupted sleep, early awakening and other insomnia symptoms affect up to 30-50% of adults [1,2]. Some studies indicate that the prevalence of insomnia symptoms increases with age [1,3], and is associated with negative effects on cognitive function [4,5]. In addition, sleep disorders are related to an increased risk of cardiovascular disease [3,6], depression [7], falls [8] and increased mortality [9].

Treatment of sleep problems in older adults can be particularly challenging for various reasons. First, sleep duration and quality are reduced with increasing age [5], but insomnia symptoms should, however, not be dismissed as just a part of normal aging [1,5]. Second, there is a higher prevalence of comorbidity and polypharmacy in the elderly [10,11], as well as issues regarding social aspects and compliance [12]. Finally, body composition and organ function change with increasing age, resulting in altered pharmacokinetics and -dynamics. This needs to be considered in order to avoid adverse drug reactions [12].

The world’s population of elderly people is predicted to almost triple in a few decades. In the Nordic countries, almost a third of the population is expected to be aged 60 years or over by 2050 [13]. Sleep problems are associated with a substantial economic burden on society [14], accounting for 6% of all contacts in Danish general practice and causing a total of 2 million consultations per year [15] in a country with only 5.8 million inhabitants [16]. Therefore, understanding the reasons for insomnia symptoms in the elderly in order to optimise the treatment is relevant from a patient oriented as well as a socioeconomic point of view.

The aim of this study is to describe reported reasons for insomnia symptoms in patients older than 65 years and analyse how these reasons are associated with prescription of sleep medication in general practice.

## Methods

### Design

An audit was conducted using a method developed by Audit Projekt Odense (APO), a part of the Research Unit for General Practice in Odense, Denmark. The method is based on ideas and experiences from the Royal College of General Practitioners’ Research Unit in Birmingham. Audit data are collected with a specifically developed and pilot-tested questionnaire. The results from the audit are subsequently discussed in seminars with the aim of improving quality and education in general practice. The method has been used several times in Denmark, as well as internationally [17].

### Setting

Data registration was carried out by general practitioners (GPs) working in the Region of Southern Denmark. In Denmark, general practice functions as the primary access to the health care system. When needed, GPs can refer to the specialised health care system, hospitals and private specialists, thus also acting as coordinators and gatekeepers ensuring optimal treatment of the citizens [18].

### Data

All GPs in the Region of Southern Denmark were invited to register all consultations regarding insomnia symptoms in an audit questionnaire provided by APO (Appendix I) during 20 weekdays in September and October 2017. Besides year of birth and sex, the questionnaire contained seven topics with 42 categorical variables in total. Each topic was to be marked with minimum one X, as its variables were exhaustive. This study comprehends the topics current course (first contact regarding insomnia symptoms), reason for insomnia symptoms (psychiatric diagnosis, life crisis, loneliness, poor sleep hygiene, somatic illness, other acknowledged reason or unknown) and prescribed medication (Z-hypnotics (zopiclone, zolpidem), benzodiazepines, mirtazapine/mianserin, other antidepressants, antipsychotics (quetiapine, Truxal/Nozinan), melatonin, sedating antihistamines (Phenergan, Postafen, Marzine), other medication for insomnia or no sleep medication). Other topics were symptoms, expectations, abuse and treatment. Inclusion criteria for this study were patients older than 65 years presenting with insomnia symptoms. Registrations with missing data for any of the included variables were excluded.

### Analyses

For clarification, two terms were created for the different groups of patients. Throughout the paper, the term ‘single-reported’ will be used for patients with only one reported reason for insomnia symptoms. For patients with two or more reported reasons the term will be ‘multiple reported’ reasons. Simple logistic regression was used to analyse the association between prescription and each of the variables (reasons for insomnia (multiple and single-reported), first contact regarding insomnia symptoms, age group and sex). The association between prescription and the interaction between all the single-reported reasons was also assessed, using multiple logistic regressions. The other variables included in the study (age, sex and current course) were not adjusted for in this analysis. All variables were dichotomised into yes (coded as 1) and no (coded as 0) and analysed using the statistical software STATA/IC 15.0 for Mac. The statistical significance level was set at *p* < 0.05.

## Results

Out of 1,123 consultations registered in the audit, a total of 405 concerned elderly patients and were included in this study. A majority (69%) of the patients were female, and a majority (82%) had previously consulted their GP regarding sleep problems. A fifth (21%) reported multiple reasons for their insomnia (Table 1).

**Table 1.** Baseline characteristics.

Baseline characteristics	
Consultations with patients aged > 65 years	*n* = 405 (100)
Sex	
Female	279 (69)
Male	126 (31)
Age, years	
66–74	170 (42)
75–84	155 (38)
≥ 85	80 (20)
First contact regarding insomnia	
Yes	73 (18)
No	332 (82)
Reported reasons for insomnia	
Psychiatric diagnosis	118 (29)
Life crisis	62 (15)
Loneliness	36 (9)
Poor sleep hygiene	38 (9)
Somatic illness	136 (34)
Other acknowledged reason	52 (13)
Unknown	62 (15)
Single or multiple reported reasons for insomnia	
Single-reported reason	320 (79)
Multiple reported reasons	85 (21)
Sleep medication prescribed	
Z-hypnotics (zopiclone, zolpidem)	197 (49)
Benzodiazepines	43 (11)
Mirtazapine/mianserin	58 (14)
Other antidepressants	30 (7)
Antipsychotics	22 (5)
Melatonin	15 (4)
Sedating antihistamines	14 (3)
Other medication for insomnia	5 (1)
No sleep medication	80 (20)

Characteristics of the registered consultations with patients aged over 65 years presenting with sleep problems; *n* (%). ‘Single-reported reason’ and ‘multiple reported reasons’ are referring to whether only one or two or more reasons for the insomnia symptoms were registered, respectively. Multiple reasons and sleep medications could be registered at one consultation, hence the total percentages in these categories exceed 100%.

### Reported reasons

**Figure 1.**
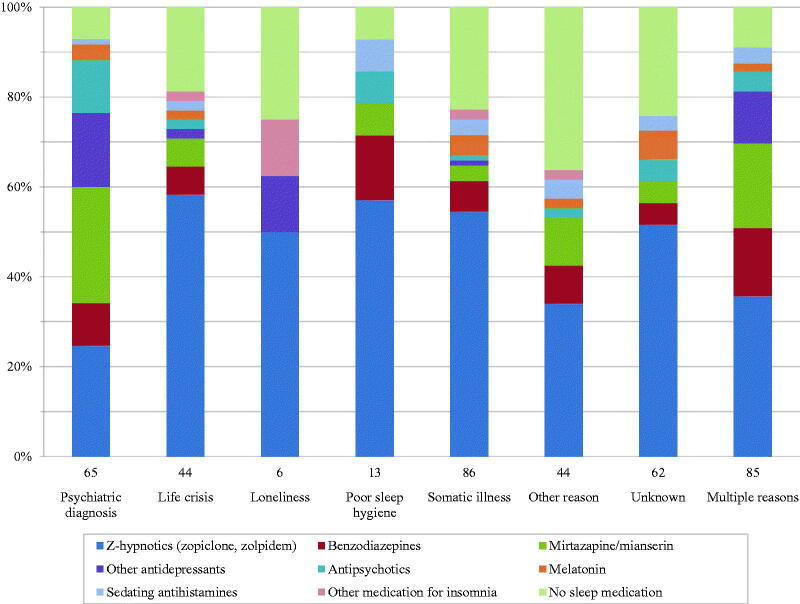
The number of consultations (*n*) is shown below the bars, divided in groups where only one (‘single-reported’) reason for the patients’ insomnia symptoms was registered. The consultations where two or more reasons were registered are shown in the bar ‘Multiple reasons’. More than one sleep medication could be registered at each consultation.

The most common reason for insomnia symptoms was somatic illness, followed by psychiatric diagnosis, both in general (*n* = 136; 34% and *n* = 118; 29%) and as single-reported reasons (*n* = 86; 21% and *n* = 65; 16%) (Table 1 and Figure 1).

Loneliness was present in 36 (9%) cases overall but seen in only 6 (1%) as single-reported reason. The same trend was seen with poor sleep hygiene, overall 38 (9%) and single-reported 13 (3%). In contrast, life crisis was almost equally common as single-reported (*n* = 44; 11%) and as overall reason (*n* = 62; 15%). Other reason than any of the stated was present in 52 (13%) cases overall and the reason was unknown in 62 (15%).

### Prescription

Sleep medication was prescribed in 80% (*n* = 325) of the consultations. The most frequent choice was Z-hypnotics (*n* = 197; 49%), followed by mirtazapine/mianserin (*n* = 58; 14%) and benzodiazepines (*n* = 43; 11%) (Table 1). Antidepressants were most commonly prescribed to patients with psychiatric diagnoses (Figure 1). The other types of medication were prescribed to 5% or fewer, respectively (Table 1). Seventy-three (18%) consultations concerned a first time visit for insomnia symptoms, of which 38 (52%) resulted in a prescription, also most commonly of Z-hypnotics (21%), mirtazapine/mianserin (12%) and benzodiazepines (8%).

Regardless of reason, it was more common to receive a prescription than not to (Figure 1). Medicine was prescribed most frequently when the reason was poor sleep hygiene (92%) or psychiatric diagnosis (91%). With somatic illness as single-reported reason, 77% received a prescription. However, this did not reach significance (odds ratio (OR) 0.92, 95% confidence interval (CI) 0.55–1.55).

For the most prevalent single-reported reasons, somatic illness and psychiatric diagnosis, the most frequently prescribed medications were Z-hypnotics (56%) and mirtazapine/mianserin (34%), respectively. When life crisis, loneliness or poor sleep hygiene were single-reported reasons, 50% or more received Z-hypnotics. Consultations not resulting in prescriptions were associated with somatic illness, loneliness, unknown and other acknowledged reason.

When using simple logistic regression (Table 2), having a psychiatric diagnosis or multiple reported reasons increased the odds for prescription (OR 4.60, CI 2.41–9.90 and OR 2.10, CI 1.03–4.28). Having a single-reported reason, other acknowledged reason or being first contact regarding insomnia symptoms decreased the odds (OR 0.48, CI 0.23–0.97; OR 0.36, CI 0.19–0.68 and OR 0.17, CI 0.10–0.30, respectively). When analysing the single-reported reasons using multiple logistic regression (Table 3), only other acknowledged reason reached significance (OR_adj_ 0.21, CI 0.09–0.52).

**Table 2.** Association between baseline characteristics and prescription.

Variables	OR	95% CI	*p* value
Sex			
Male	0.75	0.45–1.25	0.27
Female	1.33	0.80–2.23	0.27
Age, years			
66–74	0.86	0.52–1.40	0.54
75–84	1.47	0.87–2.47	0.15
≥ 85	1.14	0.41–1.33	0.31
Reported reasons for insomnia			
Psychiatric diagnosis	4.60	2.41–9.90	< 0.05
Life crisis	1.33	0.65–2.76	0.44
Loneliness	1.58	0.59–4.21	0.36
Poor sleep hygiene	1.35	0.54–3.34	0.52
Somatic illness	0.92	0.55–1.55	0.76
Other acknowledged reason	0.36	0.19–0.68	< 0.05
Unknown	0.73	0.39–1.39	0.34
Single or multiple reported reasons for insomnia			
Single-reported reason	0.48	0.23–0.97	< 0.05
Multiple reported reasons	2.10	1.03–4.28	< 0.05
Contact			
First contact regarding insomnia	0.17	0.10–0.30	< 0.05

CI: confidence interval

Crude odds ratios (OR) illustrating whether sleep medication was prescribed or not according to sex, age group, reported reason for insomnia symptoms and being first contact regarding insomnia symptoms, respectively. ‘Single-reported reason’ and ‘multiple reported reasons’ are referring to whether only one or two or more reasons for the insomnia symptoms were registered, respectively.

**Table 3.** Association between reason for insomnia symptoms and prescription.

Single-reported reason for insomnia	OR_adj_	95% CI	*p* value
Psychiatric diagnosis	1.31	0.45–3.81	0.62
Life crisis	0.52	0.19–1.39	0.19
Loneliness	0.27	0.04–1.65	0.16
Poor sleep hygiene	1.60	0.19–13.65	0.67
Somatic illness	0.44	0.19–1.01	0.05
Other acknowledged reason	0.21	0.09–0.52	< 0.05
Unknown	0.42	0.17–1.01	0.05

CI: confidence interval.

Adjusted odds ratios (OR_adj_) showing the association between sleep medication prescription and the interaction between the single-reported reasons. The other variables included in the study (age, sex and current course) are not adjusted for in this analysis. ‘Single-reported reason’ is referring to the consultations where only one reason was registered for the patients’ insomnia symptoms.

## Discussion

### Principal findings

The most frequently reported reason for insomnia symptoms among the elderly was somatic illness, followed by psychiatric diagnosis. Life crisis, other acknowledged and unknown reason were also rather common, whereas loneliness and poor sleep hygiene were reported in less than 10% of the cases, respectively. A majority (80%) of the consultations resulted in a prescription, most commonly of Z-hypnotics.

Having multiple reported reasons increased the odds for prescription (OR 2.10) whereas having only one reason decreased the odds (OR 0.48). This may be explained by GPs finding it more difficult to treat when facing multiple reasons and they were therefore more prone to prescribe medication. Also, some of the prescriptions might have been a continuation of medication initiated by other specialists in these complex cases. Psychiatric diagnosis was also significantly associated with prescription, increasing the odds with 360%. This could be explained by the fact that many drugs used for sleep disturbances are psychoactive and also used in the treatment of psychiatric illness. The other dominant reason, somatic illness, was not significantly associated with prescription. However, a majority (77%) of those with a single-reported somatic reason were prescribed sleep medication. Before prescribing to these patients, GPs should consider whether the benefits outweigh the risks and if the somatic illness is otherwise optimally treated. Being first visit regarding insomnia symptoms was negatively associated with prescription (OR 0.17), which might reflect the GPs’ awareness of guidelines and effort to avoid prescribing medication at first visit. However, it is notable that half (52%) of these elderly ‘first timers’ walked out from the consultation with a prescription, indicating that sleep medication is quite commonly initiated in the elderly. Since the audit does not cover what the other acknowledged reasons are, its significant association with prescription is difficult to discuss in more detail. The variables not reaching significance could be a result of the limited sample size, if not simply an absence of association.

### Strengths and limitations

The GPs’ familiarity with the APO method, along with the simplicity of the questionnaire, strengthens the validity and reproducibility of the collected data. Participation is voluntary and unremunerated, and the immediate recording of data minimises recall bias. The GPs receive their own and the general audit results, and are invited to subsequent re-education meetings with experts within the field. Thus, the method serves both as a provider of information as well as a tool for personal and general quality development. Furthermore, since all Danish citizens have access to free health care, the study is not limited to the individual patient’s socioeconomic status.

If sleep problems account for 6% of GP consultations [15], one could expect a higher number of registered consultations. This could indicate a lack of recording of all patients, perhaps due to high workload, forgetfulness or complex cases (where insomnia is not the main reason for contact). Also, some patients might have been registered more than once during the study period due to several consultations, thus blurring the results. This is however not assumed to be a significant issue due to the short duration of the study.

The number of consultations registered as the first regarding insomnia symptoms might be overestimated since the GPs’ knowledge about the patients could be limited. For example, patients could have consulted other GPs or had a history with insomnia years ago. Also, some prescriptions were perhaps not initiated against insomnia, but were a continuation of ongoing treatment for other conditions, such as antidepressants for depression. However, the cross-sectional nature of this study does not provide further data on the GPs’ reflections and decision-making process. If only the GPs with most time, dedication and interest in self-education chose to participate, some selection bias might be present, probably resulting in an underestimation of the prescription rate.

As this study only includes the elderly, it does not provide a comparison between younger and elderly patients and the results might not be applicable to other age groups. Treating insomnia in elderly patients is important, but can be particularly challenging due to polypharmacy, multimorbidity, compliance issues and an increased vulnerability to side effects. This study, in line with others mentioned, indicates overprescribing of sleep medication to elderly patients, while little is known about the reasons for their insomnia. Therefore, this paper is dedicated to shed light on the issues regarding insomnia in this constantly growing age group.

### Other studies

To our knowledge, few other studies have been focusing on the reasons for insomnia symptoms in older adults in general practice. Arroll *et al.* [19] also found that the most prevalent reasons for insomnia symptoms in primary care are of psychiatric and somatic origin. However, the study applies to all adults and not only to the elderly. Other studies examining the association between reasons for insomnia symptoms and prescription of sleep medication were not found.

Several studies focus on pharmacological treatment of insomnia. Schroeck *et al.* [20] concluded that Z-hypnotics can be used in short term as last-resort treatment for elderly patients with insomnia symptoms. Sedating low-dose antidepressants can be considered in comorbid depression. However, benzodiazepines and antipsychotics should be avoided due to side effects, and melatonin is not recommended due to inconsistent effect. The European Insomnia Guideline [21] agrees with these recommendations, however, accepts limited use of benzodiazepines. The American Society of Geriatrics [22] also recommends generally avoiding sedating antihistamines and benzodiazepines, and, unlike the others, avoidance of Z-hypnotics. The prescription pattern of the GPs in our study is quite in accordance with a mixture of these recommendations: Z-hypnotics were most frequently prescribed, and in case of psychiatric diagnosis, antidepressant drugs were more frequently used. Regarding the studies’ disagreement on benzodiazepines, we found that the GPs prescribed these drugs to elderly patients but less frequently than Z-hypnotics and antidepressants. Although not recommended, a small part of the consultations resulted in antihistamine, antipsychotic or melatonin prescriptions.

More than half (52%) of the first consultations regarding insomnia resulted in prescription, mostly of Z-hypnotics, antidepressants and benzodiazepines. This finding conflicts with international [21] as well as national [23] guidelines, which recommend that non-pharmacological interventions, such as cognitive behavioural therapy for insomnia (CBT-I), should be considered first-line treatment. Moreover, that medication with addictive potential, such as Z-hypnotics and benzodiazepines, should only be used after non-pharmacological methods are shown to be ineffective or unavailable and only as short-term treatment. This applies particularly to older adults, who generally are more sensitive to side effects and to whom the benefits of sedative hypnotics may not justify the risk of adverse events [24], such as cognitive impairment, delirium, falls and sedation [22]. A Norwegian study [25] also found that Z-hypnotics and benzodiazepines are prescribed too frequently to elderly patients in general practice. Furthermore, that these medications often are issued in large quantities indicating long-term use.

CBT-I is considered a safe and effective treatment for insomnia, possibly with even longer-lasting effects compared with sleep medication [26]. It has been shown to be effective in the elderly and a favourable treatment option, especially when considering the potential side effects of pharmacological treatment [27]. Furthermore, it is suggested that patients generally prefer non-pharmacological interventions over medication if given the choice [28]. Meeting the patients’ preferences in this respect could be beneficial regarding both compliance and treatment outcome.

The finding that medicine is often prescribed already during the first consultation, despite the recommendations, might be due to various reasons. GPs have suggested that they find solely advice on sleep hygiene insufficient but report a lack of knowledge and confidence in using CBT-I [29]. It might also be a question of lack of time, availability and general accessibility. However, studies have shown promising results for Internet-delivered [30] and group based [31] CBT-I, which could be potential ways to make non-pharmacological interventions for insomnia more applicable in a clinical setting. Since this study focuses on pharmacological treatment only, it is possible that non-pharmacological interventions were initiated simultaneously with the medication. Also, some patients might have been considered unable to comply with CBT-I and were therefore prescribed medication already at the first visit.

### Implications

This study contributes to the relatively sparse knowledge about why older adults experience sleep problems. In addition, it indicates that there is room for improvement regarding treatment of these patients in general practice. Sleep medication with potential serious adverse effects, especially in elderly patients, is prescribed too frequently. An effort should be made to make non-pharmacological interventions, e.g. CBT-I, more available. Organisational changes could be of value, e.g. by supporting GPs in allocating enough time for consultations regarding sleep problems. Furthermore, public education concerning sleep hygiene, the nature of insomnia and the limited benefits of sleep medication for elderly patients [24] could be beneficial.

### Perspectives

Future research should aim at investigating reasons for insomnia symptoms in the elderly in more detail, e.g. by specifying which somatic and psychiatric illnesses cause sleep problems and adjusting for prescriptions initiated by other specialists. Also, adjusting for the patients’ pre-existing diagnoses and medication would be of particular relevance in this age group, since alteration of their management could possibly diminish or even cure comorbid insomnia. Follow-up studies providing information about treatment duration and dosage would be of great value in order to assess treatment success according to both official recommendations and the individual patient. Furthermore, investigating the decision-making process of the GPs could help explain why there is still a gap between guidelines and reality.

## Conclusion

The most commonly reported reasons for insomnia symptoms in older adults were somatic illness and psychiatric disease, suggesting a high prevalence of comorbid insomnia. Having multiple reported reasons or a psychiatric diagnosis was significantly associated with greater odds for prescription of sleep medication. Regardless of the reason reported, a majority of the consultations resulted in prescription of sleep medication with potential serious adverse effects in elderly patients, indicating that there is still room for improving the management of insomnia in this age group.
